# Supplementary material for: MetaRibo-Seq measures translation in microbiomes
Source: Nat Commun. 2020 Jun 29;11:3268. doi: 10.1038/s41467-020-17081-z (PMC7324362; doi:10.1038/s41467-020-17081-z)
Supplement: Supplementary file 10 — Supplementary Data 7 [file 41467_2020_17081_MOESM10_ESM.zip › File2/Confidence_VeryHigh_Taxonomy/1545_out.krona.html]

Javascript must be enabled to view this page.

members
magnitude
magnitudeUnassigned
count
unassigned
taxon
rank

1545\_out

4

4
superkingdom
2

1
976
phylum

1
117747
class

order
200666
1

species

SRS023557\_contig\_number\_contig-100\_702.61770.61770
2044944
1

1239
phylum
3

3
class
186801

order
186802
3

family
186803
3

43996
genus
3


SRS012285\_contig\_number\_17035SRS077520\_contig\_number\_81504SRS147281\_contig\_number\_509
species
43997
3
